# CISD2 Promotes Resistance to Sorafenib-Induced Ferroptosis by Regulating Autophagy in Hepatocellular Carcinoma

**DOI:** 10.3389/fonc.2021.657723

**Published:** 2021-08-16

**Authors:** Bowen Li, Shibo Wei, Liang Yang, Xueqiang Peng, Yingbo Ma, Bo Wu, Qing Fan, Shuo Yang, Xinyu Li, Hongyuan Jin, Shilei Tang, Mingyao Huang, Hangyu Li, Jingang Liu

**Affiliations:** Department of General Surgery, The Fourth Affiliated Hospital of China Medical University, Shenyang, China

**Keywords:** hepatocellular carcinoma, sorafenib resistance, ferroptosis, CISD2, autophagy, Beclin1

## Abstract

**Purpose:**

Sorafenib is a multi-kinase inhibitor that is used as a standard treatment for advanced hepatocellular carcinoma (HCC). However, the mechanism of sorafenib resistance in HCC is still unclear. It has been shown that CISD2 expression is related to the progression and poor prognosis of HCC. Here, we show a new role for CISD2 in sorafenib resistance in HCC.

**Methods:**

Bioinformatic analysis was used to detect the expression of negative regulatory genes of ferroptosis in sorafenib-resistant samples. The concentration gradient method was used to establish sorafenib-resistant HCC cells. Western blot was used to detect the protein expression of CISD2, LC3, ERK, PI3K, AKT, mTOR, and Beclin1 in HCC samples. Quantitative real-time PCR (qPCR) was used to detect gene expression. CISD2 shRNA and Beclin1 shRNA were transfected to knock down the expression of the corresponding genes. Cell viability was detected by a CCK-8 assay. ROS were detected by DCFH-DA staining, and MDA and GSH were detected with a Lipid Peroxidation MDA Assay Kit and Micro Reduced Glutathione (GSH) Assay Kit, respectively. Flow cytometry was used to detect apoptosis and the levels of ROS and iron ions.

**Results:**

CISD2 was highly expressed in HCC cells compared with normal cells and was associated with poor prognosis in patients. Knockdown of CISD2 promoted a decrease in the viability of drug-resistant HCC cells. CISD2 knockdown promoted sorafenib-induced ferroptosis in resistant HCC cells. The levels of ROS, MDA, and iron ions increased, but the change in GSH was not obvious. Knockdown of CISD2 promoted uncontrolled autophagy in resistant HCC cells. Inhibition of autophagy attenuated CISD2 knockdown-induced ferroptosis. The autophagy promoted by CISD2 knockdown was related to Beclin1. When CISD2 and Beclin1 were inhibited, the effect on ferroptosis was correspondingly weakened.

**Conclusion:**

Inhibition of CISD2 promoted sorafenib-induced ferroptosis in resistant cells, and this process promoted excessive iron ion accumulation through autophagy, leading to ferroptosis. The combination of CISD2 inhibition and sorafenib treatment is an effective therapeutic strategy for resistant HCC.

## Introduction

Hepatocellular carcinoma (HCC) is the second leading cause of cancer death worldwide (1). Due to the late presentation of symptoms, fewer than 20% of patients presenting with HCC are candidates for potentially curative treatment, such as surgical resection, ablation, or radioactive embolization (2). Once HCC becomes advanced, there are few systemic therapeutic options for its management.

Sorafenib is the only targeted drug for the treatment of advanced HCC approved by the US Food and Drug Administration, but its efficacy as a monotherapy remains unsatisfactory, with a median overall prolonged survival benefit of only 3 months compared to placebo (3). Furthermore, primary and acquired drug resistance makes the number of HCC patients with complete response to sorafenib very low (4). In view of the emerging crisis of sorafenib resistance in HCC, further research to develop a new therapeutic strategy is urgently needed.

The redox status of cancer cells usually differs from that of normal cells (5), and the increases in antioxidant factors induce the initiation and progression of HCC (6). Ferroptosis, defined as a new programmed oxidative cell death discovered in recent years, is characterized increased ROS production *via* the Fenton reaction and the accumulation of lipid peroxidation products (7). Sorafenib can induce HCC cell ferroptosis resulting from inhibition of system $X_{C}^{-}$ followed by cellular GSH depletion (8, 9), and the induction of ferroptosis is a promising strategy to combat apoptosis-resistant HCC (10). It was previously reported that capecitabine (an anti-metabolic fluorouracil deoxynucleoside carbamate drug) can induce ferroptosis in metastatic breast cancer (11), and relatedly, capecitabine was reported to be safe and effective in HCC patients experiencing sorafenib failure (12–14).

NEET proteins are conserved proteins that retain the CDGSH iron sulfur domain (CISD) and are encoded by three genes: CISD1 encodes mitoNEET (mNT), CISD2 encodes nutrient-deprivation autophagy factor-1 (NAF-1), and CISD3 encodes MiNT (Miner2) (15). These recently discovered proteins play key roles in many processes involved in iron, iron-sulfur, and reactive oxygen homoeostasis and autophagy regulation in cells (16). The Fe-S cluster is redox active, and its biochemical properties are regulated by its redox state (17, 18). Moreover, CISD2 was shown to mediate longevity (19, 20) and to promote the development of breast cancer, cervical cancer, lung adenocarcinoma, pancreatic cancer, and prostate cancer (21–25). However, CISD2 is infrequently reported in research of liver cancer. CISD2 is a regulator of autophagy initiation under conditions of nutrient deprivation (26). Nevertheless, it is not clear whether CISD2 regulates autophagy in drug-resistant cells. More biochemical studies are needed to describe precisely how CISD2 can regulate autophagy pathways and what effects it produces in cancers.

In this study, we aimed to explore the role of CISD2 in ferroptosis in sorafenib-resistant HCC and to investigate how it affects ferroptosis by regulating autophagy. Our research provides a new strategy for targeted therapy of sorafenib-resistant HCC.

## Materials and Methods

### Cell Culture and Reagents

A normal hepatocyte line (L02) and HCC cell lines (Hep3B, HepG2, Huh7, and PLC) were purchased from Shanghai Institute of Cell Bank (Shanghai, China). Huh7 cells were cultured in Dulbecco’s modified Eagle’s medium (Gibco, Grand Island, USA) supplemented with NaHCO_3_ (1.5 g/L). HepG2 and PLC cells were grown in minimum essential medium (Gibco, Grand Island, USA) with NaHCO_3_ (1.5 g/L) and sodium pyruvate. Cells were cultured with 10% fetal bovine serum (Pansera ES, Pan biotech GmbH, Germany), penicillin (U/ml), and streptomycin (0.1 mg/ml) at 37°C in a humidified atmosphere containing 5% CO_2_. Cells were exposed to sorafenib (Solarbio, Beijing, China) for the indicated time and at the indicated concentration. Drug-resistant cell lines were established by stepwise selection of cells cultured in growth media with increasing concentrations of the drug over a period of 6 months. Erastin, staurosporine (STS), ferrostatin-1 (Fer-1), deferoxamine (DFO), ZVAD-FMK, and necrosulfonamide (NSA) were purchased from MedChemExpress (New Jersey, USA). Sorafenib and pioglitazone (Pg) were purchased from Solarbio Biotechnology Company (Beijing, China).

### HCC Patients

Human HCC tissues and adjacent normal tissues (ANTs) were collected from patients with hepatocellular carcinoma at the Fourth Affiliated Hospital of China Medical University and Liaoning Cancer Hospital, and all patients were diagnosed by pathological examination. Sample collection was approved by the research ethics committee of the Fourth Affiliated Hospital of China Medical University. Each patient provided informed consent to participate in the study. The patients had not undergone preoperative chemotherapy or radiotherapy. All collected samples were immediately frozen in liquid nitrogen and stored until subsequent analysis.

### Bioinformatics Technology

The expression levels of genes in sorafenib-resistant HCC samples from the Gene Expression Omnibus (GEO) database were obtained. Differential gene expression analysis was performed with R-based open-source software. Kaplan–Meier survival analysis was performed on patients stratified by CISD2 expression (high expression *vs* low expression) with the R “ggplot2” package. For Gene set enrichment analysis (GSEA), the RNA sequencing data for 374 samples of HCC and 50 samples of normal tissues were extracted from the Cancer Genome Atlas (TCGA) program database. In brief, the 374 patients were divided into the CISD2 high expression group and the CISD2 low expression group. We used GSEA v2.0 software for GSEA (http://software.broadinstitute.org/gsea/index.jsp). Statistical significance was evaluated by comparing the enrichment fractions from the enrichment results to obtain P values.

### Cell Viability Assays

Cell viability was assessed using a cell counting kit-8 (CCK-8) (Yeasen, Shanghai, China). Cells were seeded in 96-well plates at 5 × 10^3^ cells/well and transfected with CISD2 shRNA, Beclin1 shRNA, or the corresponding negative control shRNA (Ctrl shRNA) for 48 h. Then added with different concentrations of sorafenib or indicated concentration of erastin, STS, Fer-1, DFO, ZVAD-FMK, NSA cultured for 24 h at 37°C. After that, CCK-8 reagent was added and incubated for 2 h at 37°C. Thereafter, the absorbance was measured at 490 nm using a SpectraMax M2 microplate reader (Molecular Devices, Sunnyvale, CA, USA).

### Apoptosis Analysis

Apoptosis was detected using an FITC Annexin V/Dead Cell Apoptosis Kit (Thermo Fisher Scientific, Waltham, MA, USA). After transfected with CISD2 shRNA or Ctrl shRNA for 48 h, cells were treated with sorafenib (10 μmol) for 24 h and washed with PBS three times. Determine the cell density and dilute in 1× annexin-binding buffer to 1 × 10^6^ cells/ml, and 5 μl of FITC annexin V, 1 μl of 1× propidium iodide (PI, 100 μg/ml) were added to the suspension for 15 min in the dark. Finally, 400 µl of 1× binding buffer was added to each tube. Flow cytometry (BD LSRFortessa, Becton Dickinson, USA) was used to detect apoptosis in each group.

### Cell Proliferation Assay

Cell proliferation was evaluated by 5-ethynyl-2’-deoxyuridine (EdU) incorporation or colony formation assays. The EdU incorporation assay was performed with a Cell-Light™ EdU Apollo567 *In Vitro* Kit (RIBOBIO, Guangzhou, China). Cells in the logarithmic growth phase were seeded into 96-well plates with 1×10^5^ cells/well and cultured for 24 h. After 48 h of transfection with CISD2 shRNA and the corresponding Ctrl shRNA, cells were treated with sorafenib and cultured for 24 h. After that, 50 μmol/L EdU-containing medium was prepared, and the cells were incubated for 2 h. Then, the cells were fixed with 4% paraformaldehyde for 30 min room temperature and added 100 μl 0.5% TritonX-100. After that, cells were stained with 1×Apollo^®^ solution for 30 min room temperature and kept in the dark. Finally, DNA was stained with 1× Hoechst33342 and incubated at room temperature for 30 min. Images were acquired under an inverted fluorescence microscope. For the colony formation assay, 1×10^3^ cells were seeded per well. After 24 h, the HCC cells were treated with different concentrations. After another 2 weeks, the cells were washed with PBS and stained with 0.1% Crystal Violet Stain Solution (Yeasen, Shanghai, China).

### Quantitative Real-Time Polymerase Chain Reaction (qPCR)

After transfection with CISD2 shRNA, Beclin1 shRNA, or the corresponding Ctrl shRNA for 48 h, total RNA was extracted with TRIzol (Invitrogen, Thermo Fisher Scientific, USA). Then, a 1/5 volume of chloroform was added for extraction and centrifugation to obtain the upper clear liquid phase, and the same volume of isopropanol was then added and stored at −20°C overnight. cDNA was obtained by reverse transcription with a PrimeScript™ RT Reagent Kit (Promega, USA) in the Promega GoScript reverse transcription system (A5000). Real-time PCR analysis was performed using Promega GoTaq^®^ qPCR Master Mix in an ABI 7500 Fast Real-Time PCR System (Applied Biosystems, USA). With 18S rRNA as the internal reference, qPCR was carried out in a 20 μl reaction system. The 2^−ΔΔCt^ method was used to analyze the data. Three complex wells were set up for all reactions, and the experiment was repeated three times.

### Western Blot Assay

Cells were transfected with CISD2 shRNA, Beclin1 shRNA, or the corresponding Ctrl shRNA for 48 h. Then, cells were treated with sorafenib (10 μmol) for 24 h. RIPA buffer was used to extract the lysate from hepatocellular carcinoma cells, and the protein concentration was determined with a BCA protein assay kit (Beyotime, Shanghai). Then, buffer was added for denaturation of the proteins in the lysate. Total protein was separated by 7–15% SDS-PAGE. Electrophoresis was carried out at 80 V for 20 min and 100 V for 1 h and 30 min. Then, proteins were transferred to a polyvinylidene difluoride (PVDF) membrane at 50 V for 2 h. Then, 5% bovine serum albumin (BSA; Biosharp, Beijing, China) was used to block the membrane for 2 h. The membrane was incubated with antibodies specific for CISD2 (Proteintech, 1:2,000 dilution), LC3, ERK, p-ERK, PI3K, p-PI3K, AKT, p-AKT, mTOR, p-mTOR, Beclin1, and β-actin (all from Cell Signaling Technology, 1:1,000 dilution) overnight at 4°C. The next day, the membrane was washed three times with PBS for 10 min each. A horseradish peroxidase (HRP)-labeled goat anti-mouse antibody (Cell Signaling Technology, 1:5,000 dilution) was added and incubated at room temperature for 1 h. Then, the membrane was washed with PBS for 10 min each. Finally, immunoreactions were detected with an ECL Substrate Kit (Tanon 5200, Shanghai, China), and the experiment was repeated three times.

### RNA Interference and Gene Transfection

All plasmids were purchased from GeneChem (Shanghai, China). Cells were seeded in six-well plates at a density of 5×10^5^ cells/well. Cells were seeded, and after 24 h, when the cell density reached 50–70%, they were transfected with CISD2 shRNA, Beclin1 shRNA, or the corresponding Ctrl shRNA with Lipofectamine 3000 (Thermo Fisher Scientific, Waltham, MA, USA). The medium was changed after 6 h.

### ROS Level Assay

Reactive oxygen species (ROS) generation in HCC cells was assessed with 2’,7’-dichlorofluorescein diacetate (DCFH-DA) (Beyotime, Shanghai, China). Cells were seeded into six-well plates with 5×10^5^ cells/well, and CISD2 shRNA or Ctrl shRNA was transfected for 48 h. Then cells were treated with sorafenib or erastin for 24 h. DCFH-DA was diluted with serum-free medium at a ratio of 1:1,000, and the final concentration was 10 μmol/L. The cell culture medium was removed, and DCFH-DA was added. The cells were incubated at 37°C for 20 min. The cells were washed three times with serum-free medium. ROS levels were analyzed by flow cytometry (BD LSRFortessa, Becton Dickinson, USA) or immunofluorescence assays.

### Lipid Peroxidation Assay

Intracellular lipid peroxidation was evaluated by measuring the concentration of malondialdehyde (MDA) with a Lipid Peroxidation MDA Assay Kit (Beyotime, Shanghai, China). After transfection with CISD2 shRNA, Beclin1 shRNA, or the corresponding Ctrl shRNA for 48 h, cells were treated with sorafenib or erastin for 24 h. Then, a certain number of cells was collected, and the lysate was incubated at 4°C for 2 h and centrifuged at 12,000 × r for 10 min to obtain the supernatant for subsequent analysis. Then, 0.2 ml of MDA detection working solution was added. After mixing, the mixture was heated to 100°C or boiled in a water bath for 15 min. The absorbance was measured at 532 nm with a microplate reader. After the MDA content in the sample solution was calculated, it was normalized to the MDA content in the parental cell sample as the protein content per unit weight.

### Measurement of Iron Content

The intracellular iron content was measured by using calcein-acetoxymethyl ester (Ca-AM; AnaSpec, Campus Drive Fremont, USA). Cells were seeded into six-well plates with 5×10^5^ cells/well and cultured for 24 h. Then cells were transfected with CISD2 shRNA or the corresponding Ctrl shRNA for 48 h. After that, the cells were treated with sorafenib or erastin for 24 h. The cells were collected and cultured at 37°C at a density of 1×10^6^ cells/ml in 0.05 μmol Ca-AM for 15 min. After that, the cells were washed twice with 0.5 ml of PBS and used for flow cytometric analysis or immunofluorescence assays. Calcein was excited at 488 nm, and fluorescence was measured at 525 nm. The difference in the average fluorescence intensity reflected the iron content. A Prussian Blue Iron Stain Kit (Solarbio, Beijing, China) was used to detect trivalent iron.

### GSH Assay

Cellular GSH in HCC cell lysates was evaluated with a Micro Reduced Glutathione (GSH) Assay Kit (Solarbio, Beijing, China). Cells were seeded into six-well plates and cultured for 24 h. Then cells were transfected with CISD2 shRNA, Beclin1 shRNA, or the corresponding Ctrl shRNA for 48 h. After that, the cells were treated with sorafenib or erastin for 24 h. Approximately 1×10^6^ cells/ml of each sample was collected. First, the cells were washed twice with PBS (cells were resuspended in PBS and centrifuged at 600 × r for 10 min). A three-fold volume of the cell precipitation reagent was added to resuspend the cells, and the cells were subjected to two to three freeze-thaw cycles and centrifuged at 8,000 × r for 10 min. The supernatant was collected at 4°C for testing. Then, 20 μl of the sample, 140 μl of Reagent II, and 40 μl of Reagent III were added sequentially. After mixing, the mixture was allowed to stand for 2 min, and the absorbance (A2) was measured at 412 nm: ΔA= A2 - A1.

### Immunofluorescence Staining

Cells were seeded on the prepared cover glasses in six-well plates for 24 h and were reached to 30%. Then, Ctrl shRNA or CISD2 shRNA plasmid was transfected and cultured for 48 h. After that, cells were treated with sorafenib for 24 h. After cells were washed three times with PBS for 5 min each, cells were fixed with 4% polyoxymethylene for 15 min at room temperature and washed with PBS three times again. The cells were incubated with 1% Triton X-100 for 20 min at room temperature and washed three times with PBS for 5 min each. Then, the cells were incubated with bovine serum albumin (BSA) for 1 h in 37°C and washed with PBS three times, after which LC3-labeled goat anti-mouse antibody was added and incubated at 37°C for 2 h. Finally, DAPI was used to stain nuclei, and the cells were incubated in the dark for 5 min. PBS was used to wash the cells three times for 5 min each. The number and distribution of LC3 were observed under a fluorescence microscope.

### Transmission Electron Microscopy

By transfected CISD2 shRNA or the corresponding Ctrl shRNA for 48 h, and cells were treated with sorafenib for 24 h. The collected cells were treated with 5% glutaraldehyde and stored at 4°C overnight. Then samples went through ultracryomicrotomy to generate slices of 70 nm in thickness and staining with 20 μ 2% phosphotungstic acid for 10 min. All samples were analyzed by a H-7650 electron microscope at 100KV.

### Co-immunoprecipitation

Drug-resistant HCC cells were collected and incubated on ice for 40 min in 300 μl of lysis buffer containing a protease inhibitor. Then, the supernatant was collected, and 2 μg of the anti-CISD2 antibody, anti-Beclin1 antibody, or immunoglobulin G (Proteintech) was added and incubated overnight at 4°C. Then, 20 μl of protein A/G-agarose beads (Santa Cruz Biotechnology, Shanghai, China) was added and incubated at 4°C for 3 h with shaking. The beads were collected and washed with lysis buffer three times. Finally, 40 μl of loading buffer was added and boiled for 5 min, and western blot analysis was performed.

### Immunohistochemistry

The expression of CISD2 in human HCC tissues was observed in paraffin sections. Antigen was retrieved with citrate, and the sections were dehydrated and cleared through an alcohol gradient. The sections were stained with an immunohistochemical kit (Invitrogen, Thermo Fisher Scientific, USA) according to the manufacturer’s instructions. The sections were incubated with an anti-CISD2 primary antibody (Proteintech, 1:100) and were then restained with hematoxylin. Each slide was observed under a microscope.

### Statistical Analysis

Data are presented as the mean ± standard deviation (SD) or standard error of the mean values. The statistical significance of differences between treatment groups was assessed by using the Mann–Whitney U-test or analysis of variance (ANOVA) with the Bonferroni *post hoc* test. The statistical software IBM^®^ SPSS^®^ Statistics version 24.0 for Windows (IBM, Armonk, NY, USA) was used. Statistical significance was defined as a two-sided P value <0.05.

## Results

### CISD2 Is Upregulated in HCC Cell Lines and Related to Poor Prognosis

**Figure 1:**
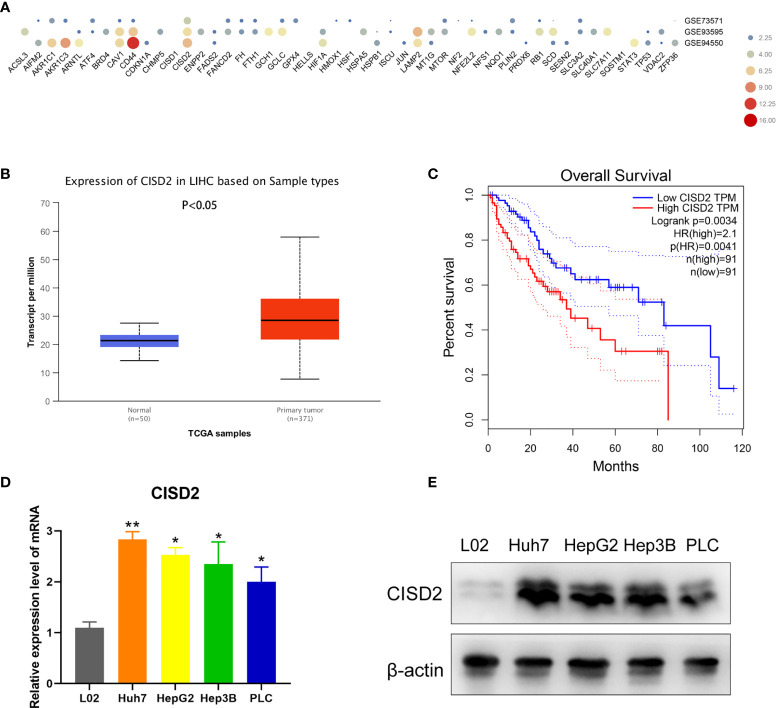
CISD2 is upregulated in HCC cell lines and related to poor prognosis. **(A)** Bioinformatics was used to detect the expression level of ferroptosis genes in gene expression omnibus (GEO) database from GSE73571, GSE93595, GSE94550. **(B)** TCGA database was used to analyze the HCC RNA-seq data of 371 tumor tissues against 50 adjacent normal tissues. **(C)** GEPIA database was used to detect the Kaplan-Meier survival analysis. **(D, E)** qPCR and western blot were used to detect the expression level of normal hepatic epithelial cell (L02) and HCC cell lines (Huh7, HepG2, Hep3B, PLC) (n=3, ^*^ *P* < 0.05, ^**^ *P* < 0.01 *versus* L02).

To explore the relationship between ferroptosis and drug resistance, we identified 49 negative regulators of ferroptosis in the ferroptosis database (http://www.zhounan.org/ferrdb/). Then, we analyzed the expression levels of genes in samples of sorafenib-resistant HCC tissue in the Gene Expression Omnibus (GEO) database (GSE73571, GSE93595, GSE94550). CISD2 was more highly expressed than the other investigated genes **(** **Figure 1A** **)**. By comparing the HCC RNA-seq data of 371 tumor tissues with that of 50 adjacent normal tissues from TCGA, we found that CISD2 was upregulated in HCC **(** **Figure 1B** **)**. In addition, Kaplan-Meier survival analysis indicated that the survival rate of patients with relatively high CISD2 expression was lower than that of patients with low CISD2 expression **(** **Figure 1C** **)**. Next, we aimed to study the potential function of CISD2 in HCC cell lines by qPCR **(** **Figure 1D** **)**. The expression of CISD2 in HCC cells (Huh7, HepG2, Hep3B, and PLC) was higher than that in normal liver cells. Western blot analysis showed that the expression level in PLC cells was relatively low and that the expression level in Huh7 cells was relatively high **(** **Figure 1E** **)**. We selected these two cell lines for subsequent research. These data suggested that CISD2 was highly expressed in HCC and correlated with the poor prognosis of HCC patients.

### Knockdown of CISD2 Reverses Sorafenib Resistance

**Figure 2:**
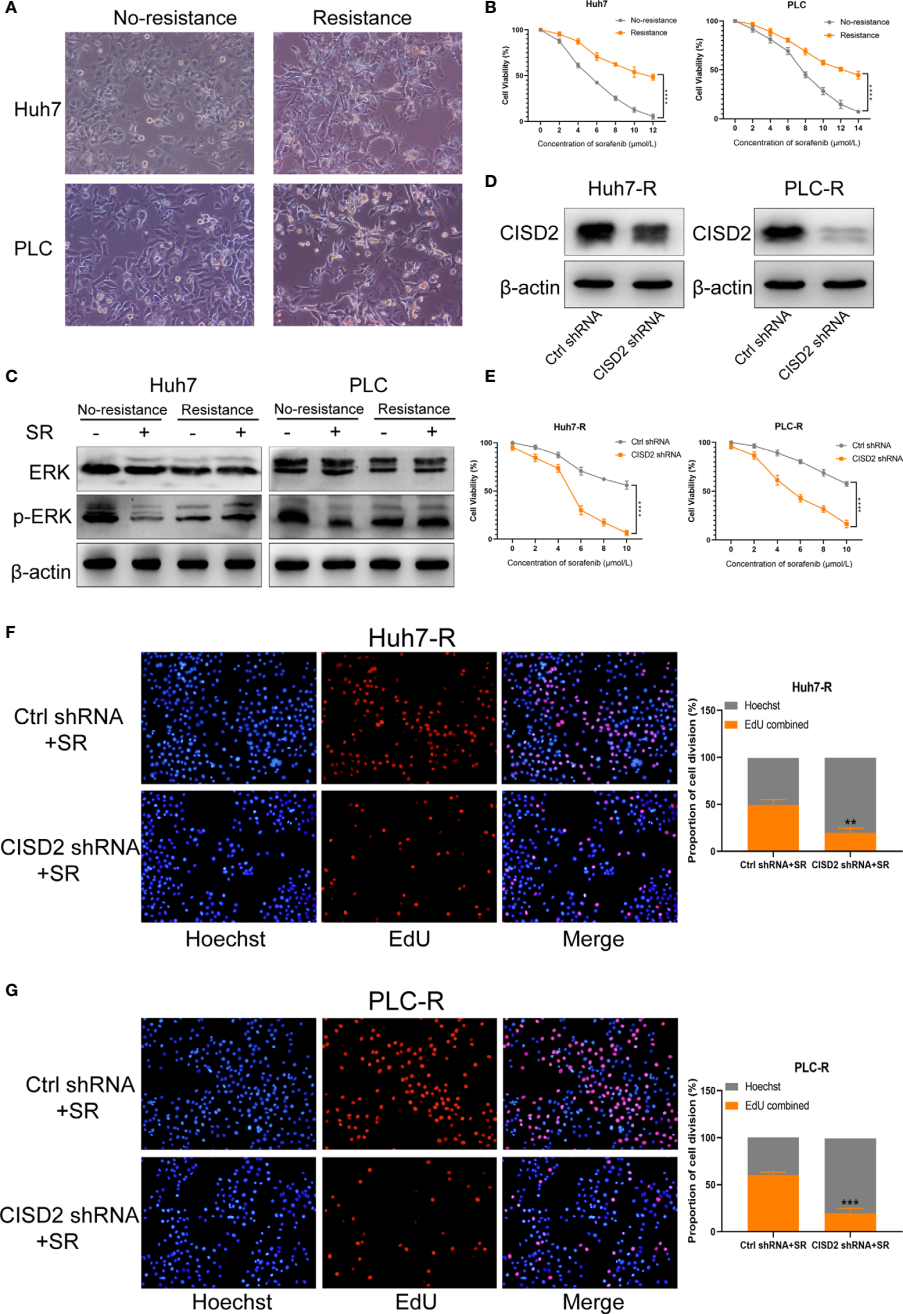
Knockdown of CISD2 reverses sorafenib resistance. **(A)** Sorafenib-resistant cells were cultured for 6 months by the concentration gradient increasing method, and the morphology of resistant cancer cells was detected. **(B)** CCK-8 method was used to detect the cell viability under treatment with different concentrations of sorafenib (n = 3, ^****^ *P* < 0.0001 *versus* No resistance). **(C)** Western blot was used to detect the expression level of ERK and p-ERK protein on treatment with sorafenib (SR, 10 μmol). **(D)** Western blot was used to detect the expression level of CISD2 under transfected CISD2 shRNA or Ctrl shRNA in Huh7-resistant cell (Huh7-R) and PLC-resistant cell (PLC-R). **(E)** CCK-8 method was used to detect the cell viability with different concentrations of sorafenib under transfected CISD2 shRNA or Ctrl shRNA (n = 3, ^****^ *P* < 0.0001 *versus* Ctrl shRNA). **(F, G)** EdU method was used to detect cell proliferation under treatment with sorafenib (10 μmol) or transfected CISD2 shRNA in resistant cells (n = 3, ^**^ *P* < 0.01, ^***^ *P* < 0.001 *versus* Ctrl shRNA+SR).

We used Huh7 and PLC cells to establish sorafenib-resistant cell lines using the concentration gradient method (from 1 to 5 μmol) over a 6-month period **(** **Figure 2A** **)**. A CCK-8 assay was used to detect the changes in cell viability under sorafenib treatment **(** **Figure 2B** **)**. The IC_50_ in Huh7 cells was 5.31 ± 0.428 μmol, while that in resistant Huh7 cells was 11.71 ± 1.775 μmol; the IC_50_ in PLC cells was 7.449 ± 0.336 μmol, while that in resistant PLC cells was 13.532 ± 1.009 μmol **(** **Supplemental Figure S1A** **)**. After sorafenib treatment at 10 μmol, the level of p-ERK in drug-resistant cells was not reduced, while the level of p-ERK in non-drug-resistant cells was reduced **(** **Figure 2C** **)**, indicating that the drug-resistant cell lines we cultured were clearly resistant. To detect the effect of CISD2 on drug-resistant cells, we transfected CISD2 shRNA to knockdown CISD2, and qPCR was used to detect the transfection efficiency **(** **Supplemental Figure S1B** **).** After that, we selected the cells exhibiting the highest transfection efficiency for western blot analysis **(** **Figure 2D** **)**. CISD2 knockdown resulted in some loss of cell viability without sorafenib treatment, but there was no significant difference compared with Ctrl shRNA (Huh7-R, *P*=0.0686; PLC-R, *P*=0.0547), and CISD2 knockdown aggravated the toxicity of sorafenib treatment at different concentrations **(** **Figure 2E** **)**. We further detected the effect of CISD2 on the proliferation of resistant HCC cells. We used an EdU incorporation assay and found that CISD2 knockdown inhibited cell division **(** **Figures 2F, G** **)**. The colony formation assay showed that CISD2 knockdown inhibited cell proliferation **(** **Supplemental Figure S1C** **)**.

### Knockdown of CISD2 Promotes Ferroptosis in Sorafenib-Resistant Cells

**Figure 3:**
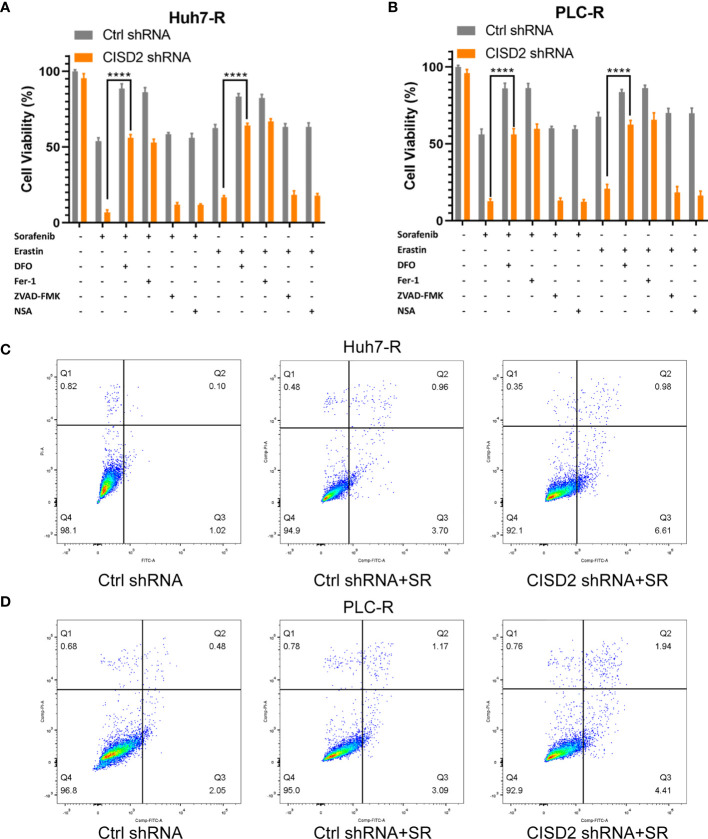
Knockdown of CISD2 mainly promotes ferroptosis and not apoptosis in sorafenib-resistant cells. **(A, B)** CCK-8 method was used to detect the cell viability under different treatment with sorafenib (10 μmol) or transfected CISD2 shRNA, and erastin (10 μmol) as positive control, with or without cell death inhibitors (Fer-1, 1 μmol; DFO, 200 μmol; ZVAD-FMK, 10 μmol) (n = 3, ^****^ *P* < 0.0001 *versus* sorafenib/erastin group). **(C, D)** Cell apoptosis was detected by flow cytometry under treatment with sorafenib (10 μmol) or transfected CISD2 shRNA (horizontal axis label FITC Annexin V and vertical axis label PI).

Inducing ferroptosis is one of the best ways to prevent cancer drug resistance, and this method is used to treat refractory high-risk neuroblastoma (27). Current studies suggest that sorafenib can induce ferroptosis in hepatocellular carcinoma cells (8). We also found that the effect of sorafenib on apoptosis was not obvious; however, the apoptosis rate was increased after STS (an apoptosis inducer) treatment **(** **Supplemental Figure S2A** **)**, and sorafenib-induced cell death was restored by co-treatment with Fer-1 or DFO (ferroptosis inhibitors) **(** **Supplemental Figure S2B** **)**. In the above study, we confirmed that CISD2 knockdown reversed sorafenib resistance. We then explored whether CISD2 knockdown can reverse sorafenib resistance by inducing ferroptosis. We used sorafenib at a higher concentration (10 μmol) to treat resistant cells. A CCK-8 assay was used to detect cell viability. The results showed that Fer-1 and DFO weakened the effects of both sorafenib and erastin in both Ctrl shRNA- and CISD2 shRNA-transfected cells. However, ZVAD-FMK (an apoptosis inhibitor) and NSA (a necroptosis inhibitor) exerted no influence on sorafenib- or erastin-induced growth inhibition **(** **Figures 3A, B** **)**. Flow cytometric analysis showed that the apoptosis induced by CISD2 knockdown was not obvious under sorafenib treatment **(** **Figures 3C, D** **)**. This finding suggests that CISD2 knockdown promotes sorafenib-induced ferroptosis in resistant HCC cells.

**Figure 4:**
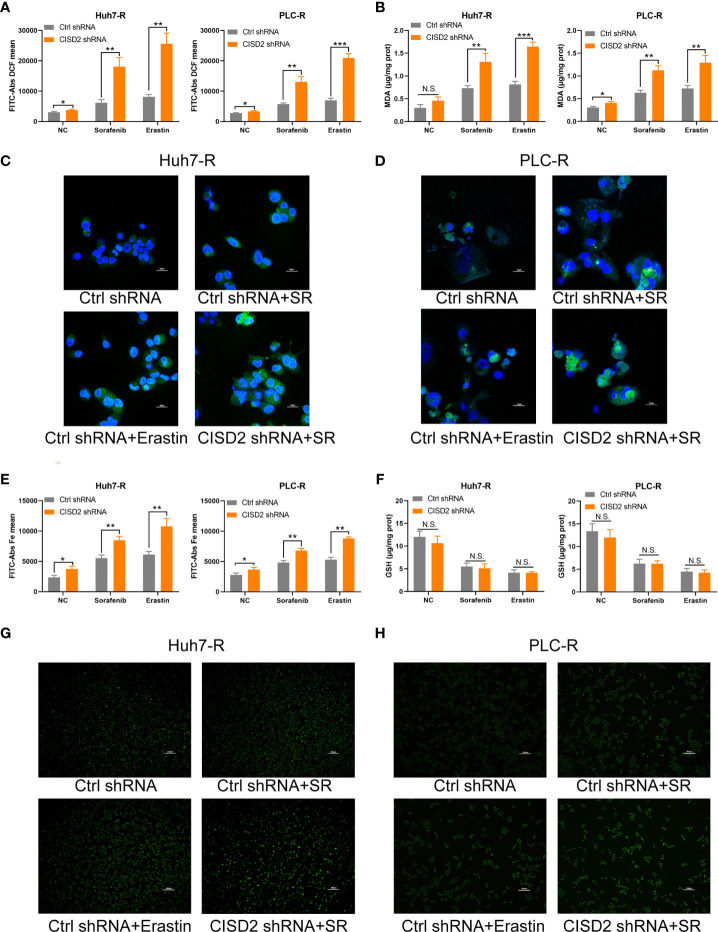
Knockdown of CISD2 promotes ferroptosis in sorafenib-resistant cells. **(A)** Flow cytometry was used to detect the expression level of ROS under treatment with sorafenib (10 μmol) or transfected CISD2 shRNA, and erastin (10 μmol) as positive control. **(B)** MDA kit was used to detect the expression level of MDA under treatment with sorafenib (10 μmol) or transfected CISD2 shRNA, and erastin (10 μmol) as positive control. **(C, D)** Immunofluorescence was used to detect the expression of ROS by adding DCFH-DA under treatment with sorafenib (10 μmol) or transfected CISD2 shRNA. **(E)** Flow cytometry was used to detect the expression level of iron ions under treatment with sorafenib (10 μmol) or transfected CISD2 shRNA, and erastin (10 μmol) as positive control. **(F)** GSH kit was used to detect the expression level of GSH under treatment with sorafenib (10 μmol) or transfected CISD2 shRNA, and erastin (10 μmol) as positive control. **(G, H)** Immunofluorescence was used to detect the expression level of iron ions under treatment with sorafenib (10 μmol) or transfected CISD2 shRNA. (n = 3, *P < 0.05, **P < 0.01, ***P < 0.001 *versus* Ctrl shRNA, N.S. means no significance).

Iron accumulation and lipid peroxidation are related to the process of ferroptosis. Thus, we detected ferrous ions, ROS, and malondialdehyde (MDA, a lipid peroxidation product). The level of ROS in the CISD2 shRNA group was higher than that in the Ctrl shRNA group under sorafenib treatment as determined by flow cytometry in Huh7-R cell (^**^ *P*=0.0028) and PLC-R cell (^**^ *P*=0.0019) **(** **Figure 4A** **)**, and the fluorescence detection also showed that knockdown of CISD2 enhanced ROS fluorescence expression **(** **Figures 4C, D** **)**. The level of MDA was increased in CISD2 shRNA group under treatment with sorafenib in Huh7-R cell (^**^ *P*=0.0069) and PLC-R cell (^**^ *P*=0.0016) **(** **Figure 4B** **)**. In addition, the content of iron ions was increased in CISD2 shRNA group under treatment with sorafenib, as shown by flow cytometry in Huh7-R cell (^**^ *P*=0.0035) and PLC-R cell (^**^ *P*=0.0026) **(** **Figure 4E** **)**, and fluorescence detection was also consistent with it **(** **Figures 4G, H** **)**. CISD2 shRNA group was highly enriched in iron ions compared with Ctrl shRNA group, as determined by high magnification fluorescence detection and Perls staining **(** **Supplemental Figure S3** **)**. However, the decrease in the GSH level induced by sorafenib appeared to not be affected by inhibition of CISD2 **(** **Figure 4F** **)**. This finding implies that inhibition of GSH itself might be less effective in the context of ferroptosis induction without iron accumulation. These results suggest that CISD2 knockdown can promote the sensitivity of drug-resistant cells to sorafenib and lead to ferroptosis.

### Knockdown of CISD2 Promotes Autophagy in Sorafenib-Resistant Cells

**Figure 5:**
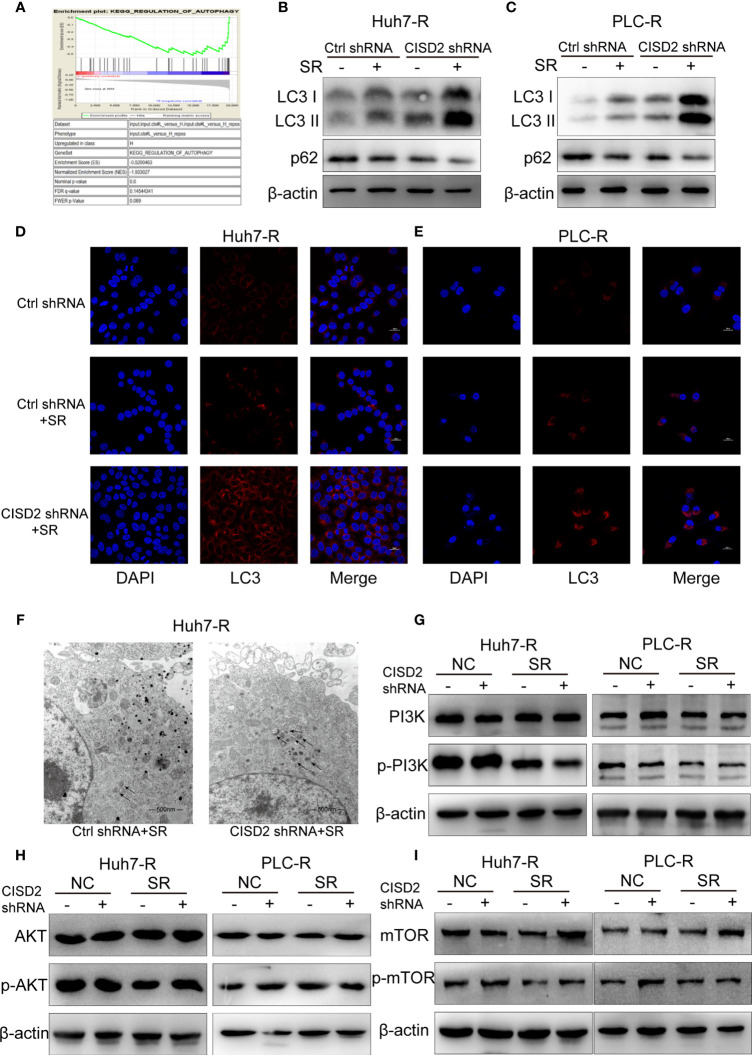
Knockdown of CISD2 promotes autophagy in sorafenib-resistant cells. **(A)** GSEA was used to detect the function of CISD2. **(B, C)** Western blot was used to detect the expression level of LC3 and p62 under treatment with sorafenib (10 μmol) or transfected CISD2 shRNA in resistant cells. **(D, E)** Immunofluorescence was used to detect the expression of LC3 under treatment with sorafenib (10 μmol) or transfected CISD2 shRNA. **(F)** Transmission electron microscopy was used to detect the number of autophagosomes under treatment with sorafenib (10 μmol) or transfected CISD2 shRNA. **(G–I)** Western blot was used to detect the expression level of PI3K, p-PI3K, AKT, p-AKT, mTOR, p-mTOR under treatment with sorafenib (10 μmol) or transfected CISD2 shRNA.

To understand the possible mechanism of CISD2, we conducted GSEA on database data for liver cancer samples. The results showed that the correlation between CISD2 and autophagy was the second highest and that there was a negative regulatory relationship between CISD2 and autophagy **(** **Figure 5A** **)**. After that, we found that the autophagy level was increased when CISD2 was knocked down. Western blot analysis showed that the LC3 I converted to LC3 II and the expression level of p62 decreased gradually **(** **Figures 5B, C** **)**. Immunofluorescence staining showed that the number of LC3 spots was increased after CISD2 knockdown **(** **Figures 5D, E** **)**. And transmission electron microscopy showed that CISD2 knockdown promoted the increase of autophagosomes **(** **Figure 5F** **)**. Studies have shown that the mTOR pathway plays an important role in the formation of autophagosomes and that the mTOR pathway is the convergence point of upstream AMPK and PI3K/Akt signal transduction (28). When the mTOR pathway is inhibited, autophagy can be activated. We used western blot to evaluate the expression of key proteins in the classic autophagy mTOR pathway. However, the levels of p-PI3K, p-AKT, and p-mTOR did not change after CISD2 knockdown **(** **Figures 5G–I** **)**. The results showed that CISD2 knockdown had no effect on the mTOR pathway.

### Inhibiting Autophagy Alleviates Ferroptosis in Sorafenib-Resistant Cells With CISD2 Knockdown

**Figure 6:**
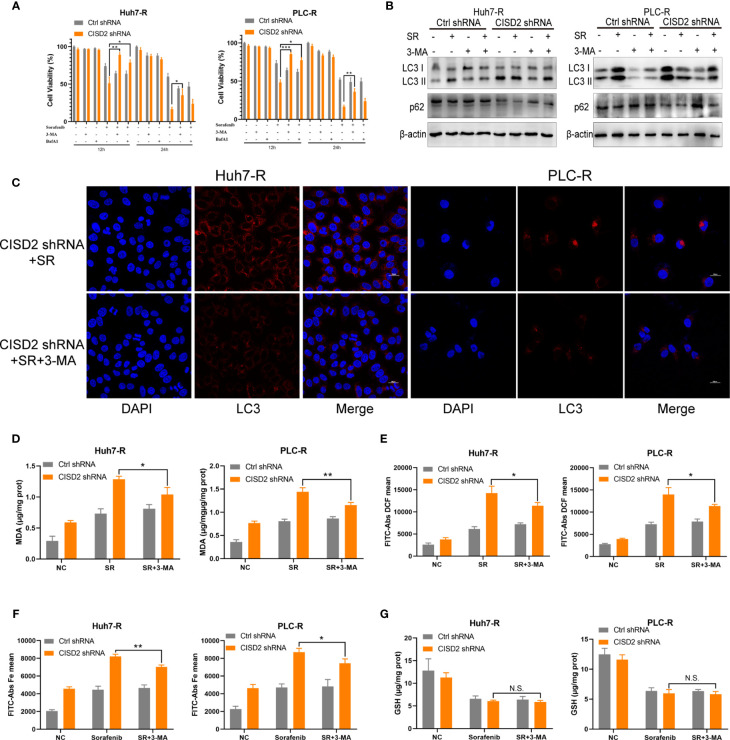
Inhibiting autophagy alleviates ferroptosis in sorafenib-resistant cells with CISD2 knockdown. **(A)** CCK-8 was used to detect the cell viability under treatment with sorafenib (10 μmol), 3-MA (1 mmol), BafA1 (20 nmol), or transfected CISD2 shRNA (n = 3, ^*^ *P* < 0.05, ^**^ *P* < 0.01, ^***^ *P* < 0.001 *versus* 3-MA treatment or BafA1). **(B)** Western blot was used to detect the expression level of LC3, p62 under treatment with sorafenib (10 μmol), 3-MA (1 mmol), or transfected CISD2 shRNA in resistant cells. **(C)** Immunofluorescence was used to detect the expression level of LC3 under treatment with sorafenib (10 μmol), 3-MA (1 mmol), or transfected CISD2 shRNA. **(D)** MDA kit was used to detect the expression level of MDA under treatment with sorafenib (10 μmol), 3-MA (1 mmol), or transfected CISD2 shRNA (n = 3, ^*^ *P* < 0.05, ^**^ *P* < 0.01 *versus* 3-MA treatment). **(E)** Flow cytometry was used to detect the expression level of ROS under treatment with sorafenib (10 μmol), 3-MA (1 mmol), or transfected CISD2 shRNA (n = 3, ^*^ *P* < 0.05 *versus* 3-MA treatment). **(F)** Flow cytometry was used to detect the expression level of iron ions under treatment with sorafenib (10 μmol), 3-MA (1 mmol), or transfected CISD2 shRNA (n = 3, ^*^ *P* < 0.05, ^**^ *P* < 0.01 *versus* 3-MA treatment). **(G)** GSH kit was used to detect the expression level of GSH under treatment with sorafenib (10 μmol), 3-MA (1 mmol), or transfected CISD2 shRNA (n=3, N.S. means no significance).

Studies have shown that autophagy plays an important role in the occurrence of ferroptosis (29–31). We thus sought to further verify whether autophagy is involved in the ferroptosis induced by CISD2 knockdown. We used the autophagy inhibitors bafilomycin A1 (BafA1) and 3-methyladenine (3-MA) separately as an autophagy initiation inhibitor and a lysosomal inhibitor, respectively, to detect autophagy. We then used CCK-8 to evaluate cell viability after 12 or 24 h of treatment. The results showed that both 3-MA and BafA1 inhibited cell death. In addition, 3-MA played a stronger role in the setting of CISD2 knockdown, and we chose it for the next experiment **(** **Figure 6A** **)**. Western blot analysis showed that after treatment with 3-MA, the change from LC3 I to LC3 II was partially decreased **(** **Figure 6B** **)**, and the number of fluorescent LC3 puncta was reduced **(** **Figure 6C** **)**. The expression levels of MDA and ROS in drug-resistant cells in the 3-MA treatment group were decreased **(** **Figures 6D, E** **)** and that iron ions were also decreased under co-treatment with 3-MA **(** **Figure 6F** **)**. The GSH level in sorafenib-resistant cells was not affected by treatment with 3-MA **(** **Figure 6G** **)**. These results suggest that inhibiting autophagy alleviates ferroptosis partially in the setting of CISD2 knockdown. In summary, CISD2 mediates cellular resistance to sorafenib-induced ferroptosis by regulating autophagy.

### Inhibition of CISD2 Promotes Ferroptosis Regulated by Beclin1 in Sorafenib-Resistant Cells

**Figure 7:**
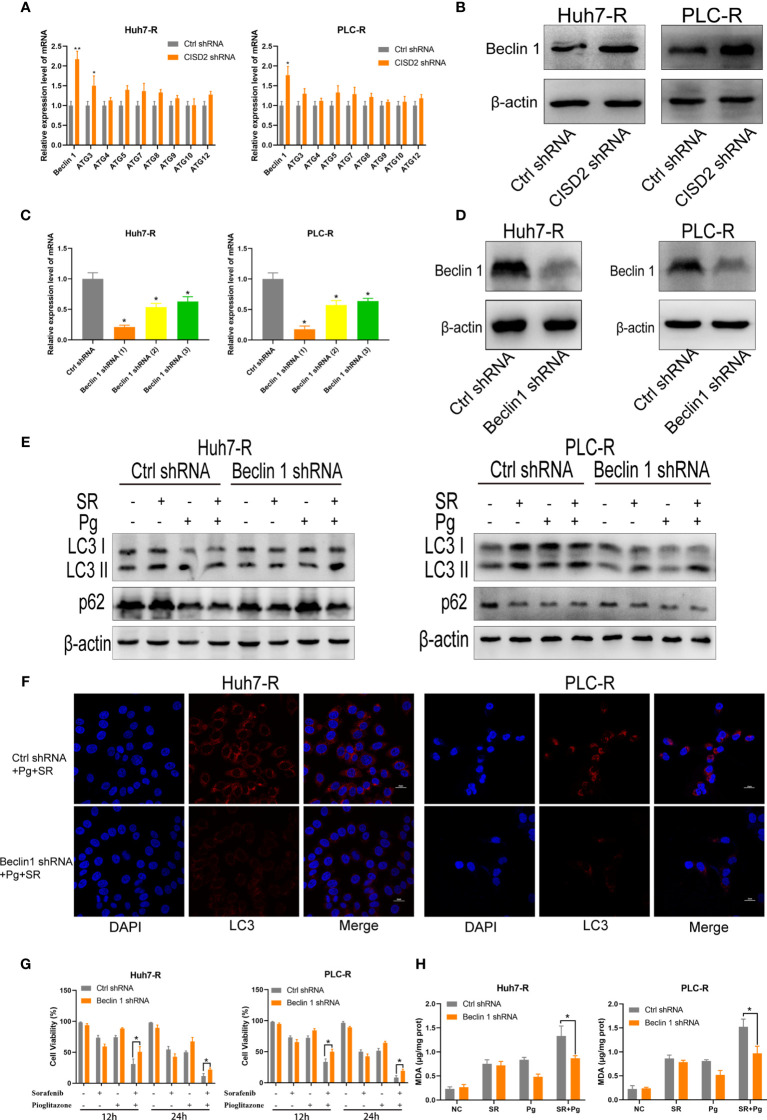
Inhibition of CISD2 promotes ferroptosis regulated by Beclin1 in sorafenib-resistant cells. **(A)** qPCR was used to detect the autophagy-related gene expression under transfected CISD2 shRNA (n = 3, ^*^ *P* < 0.05, ^**^ *P* < 0.01 *versus* Ctrl shRNA). **(B)** Western blot was used to detect the expression level of Beclin1 under transfected CISD2 shRNA. **(C, D)** qPCR and western blot were used to detect the Beclin1 expression under transfected Beclin1 shRNA (n = 3, ^*^ *P* < 0.05 *versus* Ctrl shRNA). **(E)** Western blot was used to detect the expression level of LC3, p62 under treatment with sorafenib (10 μmol), Pg (10 µmol), or transfected Beclin1 shRNA. **(F)** Immunofluorescence was used to detect the expression of LC3 under different treatment with sorafenib (10 μmol), Pg (10 µmol), or transfected Beclin1 shRNA. **(G)** CCK-8 was used to detect the cell viability under treatment with sorafenib (10 μmol), Pg (10 µmol), or transfected Beclin1 shRNA (n = 3, ^*^ *P* < 0.05 *versus* Ctrl shRNA). **(H)** MDA kit was used to detect the expression of MDA under treatment with sorafenib (10 µmol), Pg (10 µmol), or transfected Beclin1 shRNA (n = 3, ^*^ *P* < 0.05 *versus* Ctrl shRNA).

**Figure 8:**
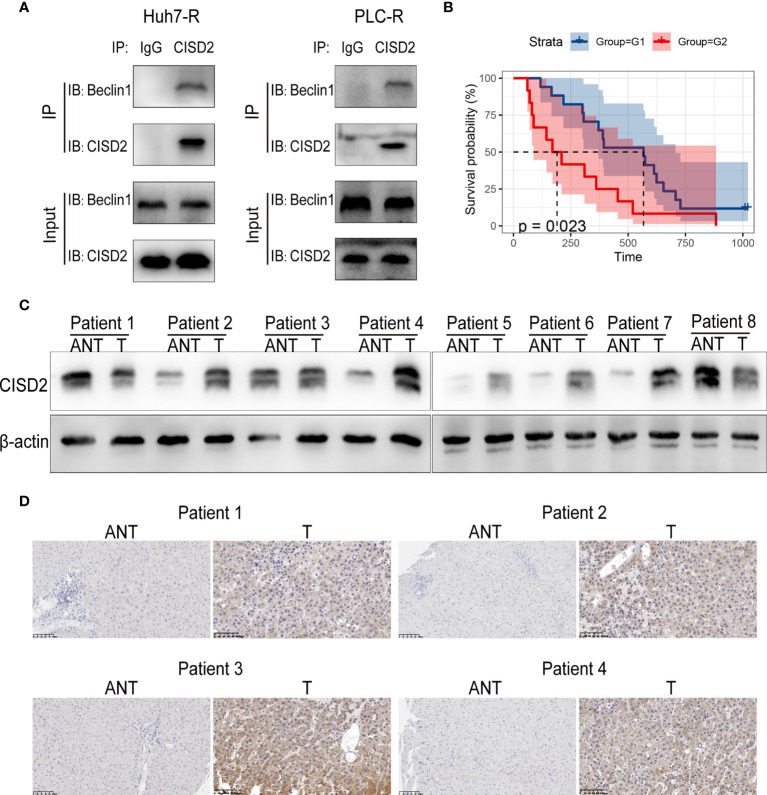
Correlation between CISD2 expression and the survival of HCC patients. **(A)** Co-immunoprecipitation of endogenous CISD2 and Beclin1 in HCC resistant cells. **(B)** Correlation between CISD2 expression and survival analysis of HCC patients (G1=CISD2 low expression group, G2=CISD2 high expression group). **(C)** Western blot was used to detect the expression level of CISD2 in HCC tissues (T) and adjacent normal tissues (ANT). **(D)** Immunohistochemistry was used to detect the expression level of CISD2 in HCC tissues and adjacent normal tissues.

To explore the regulatory target of CISD2 in autophagy, we evaluated the expression of the autophagy-related genes Beclin1, ATG3, ATG4, ATG5, ATG7, ATG9, ATG10, and ATG12 after knockdown of CISD2. The results showed that among these genes, Beclin1 was the most upregulated **(** **Figure 7A** **)**. After that, we used western blot to confirm that CISD2 knockdown promoted Beclin1 expression **(** **Figure 7B** **)**. Therefore, we explored whether CISD2 affects ferroptosis by regulating Beclin1. We transfected resistant cells with Beclin1 shRNA to knockdown Beclin1, and the knockdown efficiency was determined by qPCR and western blot analysis **(** **Figures 7C, D** **)**. In order to further explore, we used Pg as a CISD2 inhibitor to treat resistant HCC cells. After co-treatment with sorafenib and Pg, the level of LC3 was increased but was decreased by co-treatment with Beclin1 shRNA **(** **Figure 7E** **)**. The immunofluorescence results showed that LC3 spots increased after sorafenib and Pg co-treatment but decreased after Beclin1 knockdown **(** **Figure 7F** **)**. These results showed that Beclin1 knockdown partially inhibited autophagy. After that, we evaluated cell viability by CCK-8. The results showed that Beclin1 knockdown increased cell viability in sorafenib and Pg co-treatment group **(** **Figure 7G** **)**. We further examined the changes in MDA, the key indicator of ferroptosis. The results showed that knockdown of Beclin1 decreased the MDA level in sorafenib and Pg co-treatment group **(** **Figure 7H** **)**. After that, we found by co-immunoprecipitation experiments that CISD2 can bind Beclin1 **(** **Figure 8A** **)**. In conclusion, CISD2 promotes resistance to sorafenib-induced ferroptosis by regulating Beclin1 in HCC cells.

### Correlation Between CISD2 Expression and the Survival of HCC Patients

We then sought to evaluate the correlation between CISD2 expression and the survival prognosis of HCC patients. We divided patients into high and low CISD2 expression groups and found that the survival time of patients with high CISD2 expression was lower than that of patients with low CISD2 expression (*P*=0.023) **(** **Figure 8B** **)**. Western blot analysis showed that CISD2 expression in HCC tissue was higher than that in adjacent normal tissue **(** **Figure 8C** **)**. The immunohistochemical results showed that CISD2 was more highly expressed in HCC tissues than in adjacent normal tissues and was mainly localized in the cytoplasm **(** **Figure 8D** **)**. In summary, CISD2 is highly expressed in HCC patients and is associated with poor prognosis.

## Discussion

In the past few decades, although a large number of studies have been carried out to improve the efficacy of anticancer drugs by overcoming chemotherapeutic resistance, it is still a major clinical challenge in the treatment of HCC (32). Sorafenib, as the first-line drug for the treatment of advanced liver cancer, is often due to the drug resistance of liver cancer, with low response rate and poor survival and prognosis effect of patients (33). It is necessary to study the mechanism of resistance and further therapy.

The main mechanism of drug therapy is induction of apoptosis. However, cancer cells can usually develop mechanisms to prevent apoptosis by acquiring a drug resistance phenotype and upregulating pro-survival signals (34). It is necessary to explore other forms of cell death to solve the problem of drug resistance. In recent years, studies have shown that the induction of ferroptosis in cancer cells has become a new treatment strategy, and its clinical application in cancer treatment is anticipated to be promoted (35).

Currently, some clinical drugs, such as sorafenib, sulfasalazine, lanperisone, acetaminophen, and cisplatin, can induce ferroptosis in several types of cancer cells, supporting the feasibility of exploiting ferroptosis for the treatment of drug-resistant cancers (36, 37). Genetic silencing of cystine/glutamate-induced ferroptosis in resistant head and neck cancer (HNC) cells enhanced sensitivity to cisplatin (38). Furthermore, ferroptosis inducers (FINs) were able to sensitize zero-valent iron nanoparticle (ZVI NP)-resistant cancer cells to become treatable without damaging non-malignant cells (39). However, tumor cells can also resist ferroptosis through the regulation of internal antioxidant factors, thus promoting drug resistance (40). Activation of the nuclear factor erythroid-2-related factor 2 (NRF2)-antioxidant response element (ARE) pathway contributes to artesunate (a ferroptosis inducer) resistance in cisplatin-resistant head and neck cancer cells, but genetic silencing of NRF2 or trigonelline reverses artesunate resistance in cisplatin-resistant HNC cells *in vitro* and *in vivo* (41). Metallothionein (MT)-1G, a protein transcribed from NRF2, can also inhibit ferroptosis and promote sorafenib resistance in HCC (42). Therefore, understanding homoeostasis and utilizing the iron dependence of ROS may be a new anticancer treatment strategy for drug resistance in human liver cancer. Our study confirmed that sorafenib induced the HCC cell ferroptosis, further supporting the research of sorafenib-induced ferroptosis in other studies (8, 9). Therefore, it may be an effective strategy for the treatment of HCC by inducing ferroptosis.

NEET proteins are involved in regulating iron and reactive oxygen species in cancer cells (15, 43) and promote the proliferation of breast cancer cells (21). CISD2, a member of the NEET family, can promote the invasion and migration of pancreatic cancer cells and promote the tumorigenesis and poor prognosis of lung cancer (23, 24). Regarding drug resistance, CISD2 has been identified as a novel biomarker of sulfasalazine resistance, and its inhibition promotes the sensitivity of head and neck cancer cells to sulfasalazine-induced ferroptosis by increasing the accumulation of iron oxide and lipid ROS in mitochondria (44). However, the specific mechanism of CISD2 in drug resistance of HCC has not been reported. Our study revealed the mechanism of resistance to sorafenib-induced ferroptosis related to CISD2 expression. Silencing CISD2 sensitized resistant HCC cells to sorafenib-induced ferroptosis. Therefore, CISD2 was identified as a new biomarker for resistance to sorafenib-induced ferroptosis for the first time. Our results highlight CISD2 as a candidate target for modulating ferroptosis in HCC cells.

At present, the researches on ferroptosis-antagonizing tumor resistance mainly focus on the inhibition of ferroptosis defense system, such as GPX4. However, it has been reported that the core negative regulatory genes of ferroptosis include not only GPX4 but also ferroptosis suppressor protein 1 (FSP1), dihydroorotate dehydrogenase (DHODH), and others (45–47). It is a challenge to antagonize tumor resistance. However, regulating iron ions to promote ferroptosis can avoid this problem. Its purpose is more unified, that is, to promote the increase of iron ion expression in tumor cells. And to explore the regulation of iron ions to promote ferroptosis is expected to become an effective strategy for the treatment of tumor resistance (48). Autophagy, as a metabolic process invoked to cope with environmental stress and maintain homoeostasis, is of great importance for the development of tumor cells. However, there are different views on the role of autophagy in tumor cells. Currently, it is generally believed that autophagy has dual effects in the early stage of tumor occurrence and after tumor formation. On the one hand, autophagy can control the proliferation of tumor cells and inhibit angiogenesis to exert an anticancer effect (49); on the other hand, autophagy can improve the stress resistance ability of tumor cells to facilitate their survival and therapeutic resistance (50, 51). With the improved understanding of autophagy in tumor research, clinical adjuvant therapies targeting autophagy have been evaluated (52). However, the therapeutic effect and the selection of the target are still controversial.

In addition, autophagy plays an important role in the induction of ferroptosis by ferritin degradation and leads to an increase in the cellular labile iron pool, which induces oxidative stress during the occurrence of ferroptosis (29, 30). In this process, inhibition of system $X_{C}^{-}$ by ferroptotic agents (e.g., erastin and sorafenib) induces the endoplasmic reticulum (ER) stress response (9). A sustained ER stress-activated unfolded protein response (UPR) can ultimately promote cytotoxic autophagy in cancer cells (53). However, only when autophagy reaches a certain intensity does it trigger ferroptosis. Disruption of intracellular redox homoeostasis during the ferroptosis process causes mitochondrial damage and may promote the subsequent initiation of autophagy, and these events may act as a feedback loop to further induce ferroptosis until cell death occurs. Formosanin C (FC) has chemotherapeutic potential against apoptosis-resistant HCC with higher NCOA4 expression *via* ferritinophagy (10). Inhibition of autophagy and ferritinophagy reduces HCC sensitivity to sorafenib or erastin by inactivating ferroptosis (54). Carbonic anhydrase 9 (CA9) confers resistance to ferroptosis/apoptosis in malignant mesothelioma cells, and inhibition of CA9 promotes mitochondrial fission and autophagy with increased levels of catalytic Fe^2+^, peroxides, mitochondrial $O_{2}^{-}$, and lipid peroxidation (55). Since many studies have shown that autophagy can induce ferroptosis, the level of autophagic activity may play an important role in determining the target of anticancer drugs in tumor cells. In our study, we found that CISD2 inhibition promoted autophagy in sorafenib-resistant HCC cells and increased the iron content. However, ferroptosis was reduced obviously after early inhibition of autophagy. Therefore, our study showed that ferroptosis promoted by CISD2 inhibition is mainly regulated by autophagy.

**Figure 9:**
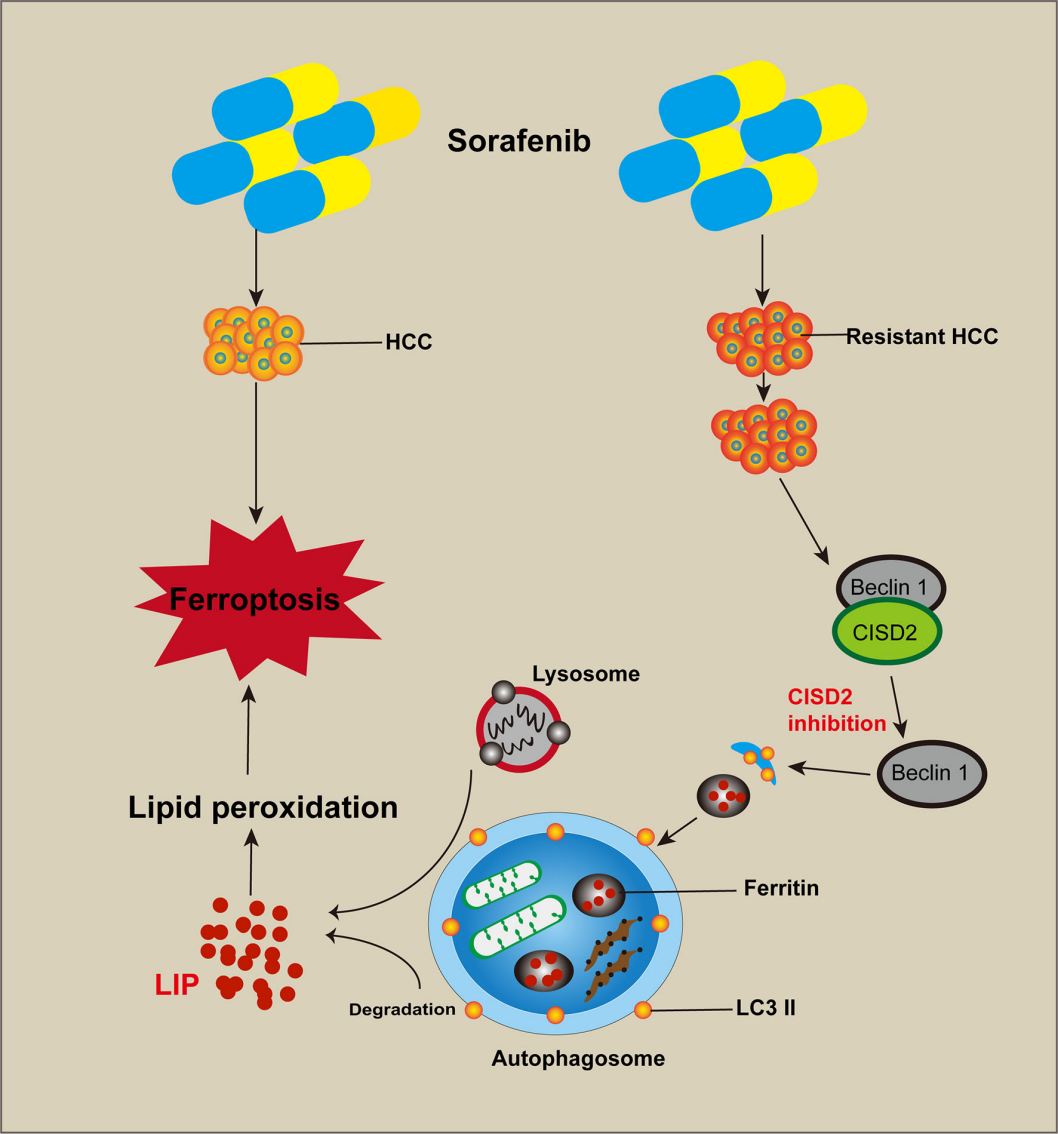
Cartoon diagram of CISD2 promotes resistance to sorafenib-induced ferroptosis by regulating autophagy in hepatocellular carcinoma. Inhibition of CISD2 restored sorafenib-induced ferroptosis and reversed drug resistance, and these effects were related to the inhibition of CISD2-promoted autophagy. Through this process, the excessive activation of autophagy promoted the release of iron ions and induced lipid peroxidation to promote ferroptosis.

In further exploration of autophagy, we found that the expression of Beclin1 was increased after autophagy was promoted by inhibiting CISD2. Studies have shown that CISD2 can bind Beclin1 in the endoplasmic reticulum and play a regulatory role in autophagy initiation (26). CISD2 interacts with BCL-2 at the ER and affects its interaction with the tumor suppressor Beclin1 (56). Beclin1 plays an important role in the regulation of autophagy and apoptosis. For example, Beclin1 interacts with class III PI3Ks to promote the induction of autophagy (57). In addition, Beclin1 directly blocks the activity of system $X_{C}^{-}$ by binding with its core component solute carrier family 7 member 11 (SLC7A11) and plays an unprecedented role in promoting ferroptosis (58). While autophagy inhibition by 3-MA or Beclin1 knockdown was partially protective against CISD2 loss of function mediated cell death, these treatments did not completely inhibit autophagy. Interestingly, sorafenib seems to override the effect of autophagy inhibition by 3-MA or Beclin1 knockdown in the absence of CISD2 function. In our study, we found that inhibition of CISD2 induces ferroptosis, which can be suppressed by silencing Beclin1, and further experiments verified that CISD2 can bind Beclin1 **(** **Figure 9** **)**. Our study revealed for the first time that autophagy promotes ferroptosis in drug-resistant cells and reverses the phenomenon of drug resistance. Our findings can provide a reference for targeted therapy of drug-resistant cancers. However, autophagy also exerts its antitumor effect through a previously unknown mechanism, and we suggest that controlling the intensity of autophagy to induce ferroptosis may be a potential treatment strategy for resistant cancers.

## Conclusions

In conclusion, the results of this study showed that CISD2 was highly expressed and related to sorafenib resistance. Inhibition of CISD2 restored sorafenib-induced ferroptosis and reversed drug resistance, and these effects were related to the inhibition of CISD2-promoted autophagy. Through this process, the excessive activation of autophagy promoted the release of iron ions and induced lipid peroxidation to promote ferroptosis. Our study provides a reference for targeted therapy of drug-resistant tumors with ferroptosis.

## Data Availability Statement

Publicly available datasets were analyzed in this study. This data can be found here: http://software.broadinstitute.org/gsea/index.jsp.

## Ethics Statement

The studies involving human participants were reviewed and approved by Ethics Committee of the Fourth Affiliated Hospital of China Medical University. The patients/participants provided their written informed consent to participate in this study.

## Author Contributions 

BL performed the experiment and wrote the manuscript. BW contributed to the analysis of bioinformatics. LY, XP, YM, QF, SW, HJ, SY, XL, MH, and ST performed reagent preparation. HL and JL conceived the study and reviewed this article. All authors contributed to the article and approved the submitted version.

## Funding

This research was supported by the National Natural Science Foundation of China (No. 81472302, No. 81871983, No. 82003040), Natural Science Foundation of Liaoning Province (LQNK201719), and Natural Science Foundation of Liaoning Province (2020-BS-103).

## Conflict of Interest

The authors declare that the research was conducted in the absence of any commercial or financial relationships that could be construed as a potential conflict of interest.

## Publisher’s Note

All claims expressed in this article are solely those of the authors and do not necessarily represent those of their affiliated organizations, or those of the publisher, the editors and the reviewers. Any product that may be evaluated in this article, or claim that may be made by its manufacturer, is not guaranteed or endorsed by the publisher.

## Supplementary Material

Supplementary Figure 1 **(A)** The IC_50_ of sorafenib resistant HCC. **(B)** qPCR was used to detected the expression level of CISD2 under transfected CISD2 shRNA. **(C)** Clone formation assay was used to detect the proliferation of resistant cells under treatment with sorafenib (10 μmol) or transfected CISD2 shRNA.

Supplementary Figure 2 **(A)** Flow cytometry was used to detect the apoptosis level of HCC cells under treatment with sorafenib (5 μmol) or STS (0.5 μmol). **(B)** CCK-8 method was used to detect the cell viability under treatment with sorafenib (5 μmol), erastin (5 μmol) with or without cell death inhibitors (Fer-1, 1 μmol; DFO, 200 μmol; ZVAD-FMK, 10 μmol) for 24 h (n=3, ^*^ *P*<0.05 *versus* NC group).

Supplementary Figure 3 Inverted fluorescence and Perls stain were used to detect the expression level of iron ions under treatment with sorafenib (10 μmol), erastin (10 μmol), or transfected CISD2 shRNA.
