# Supplementary figures and images for: CISD2 Promotes Resistance to Sorafenib-Induced Ferroptosis by Regulating Autophagy in Hepatocellular Carcinoma
Source: Front Oncol. 2021 Aug 16;11:657723. doi: 10.3389/fonc.2021.657723 (PMC8415543; doi:10.3389/fonc.2021.657723)

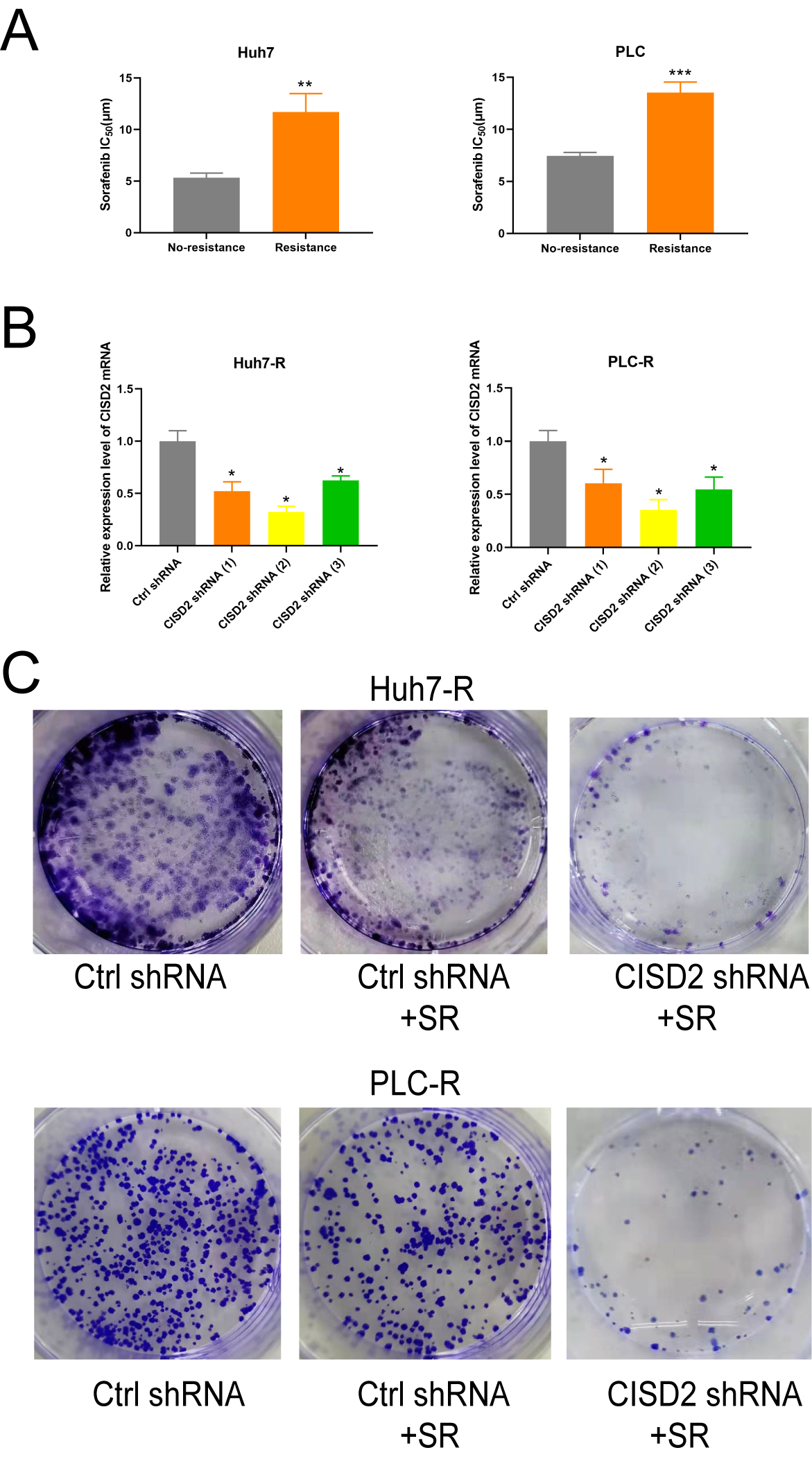

Supplement: Supplementary Figure 1 — (A) The IC50 of sorafenib resistant HCC. (B) qPCR was used to detected the expression level of CISD2 under transfected CISD2 shRNA. (C) Clone formation assay was used to detect the proliferation of resistant cells under treatment with sorafenib (10 μmol) or transfected CISD2 shRNA. [file Image_1.tif]

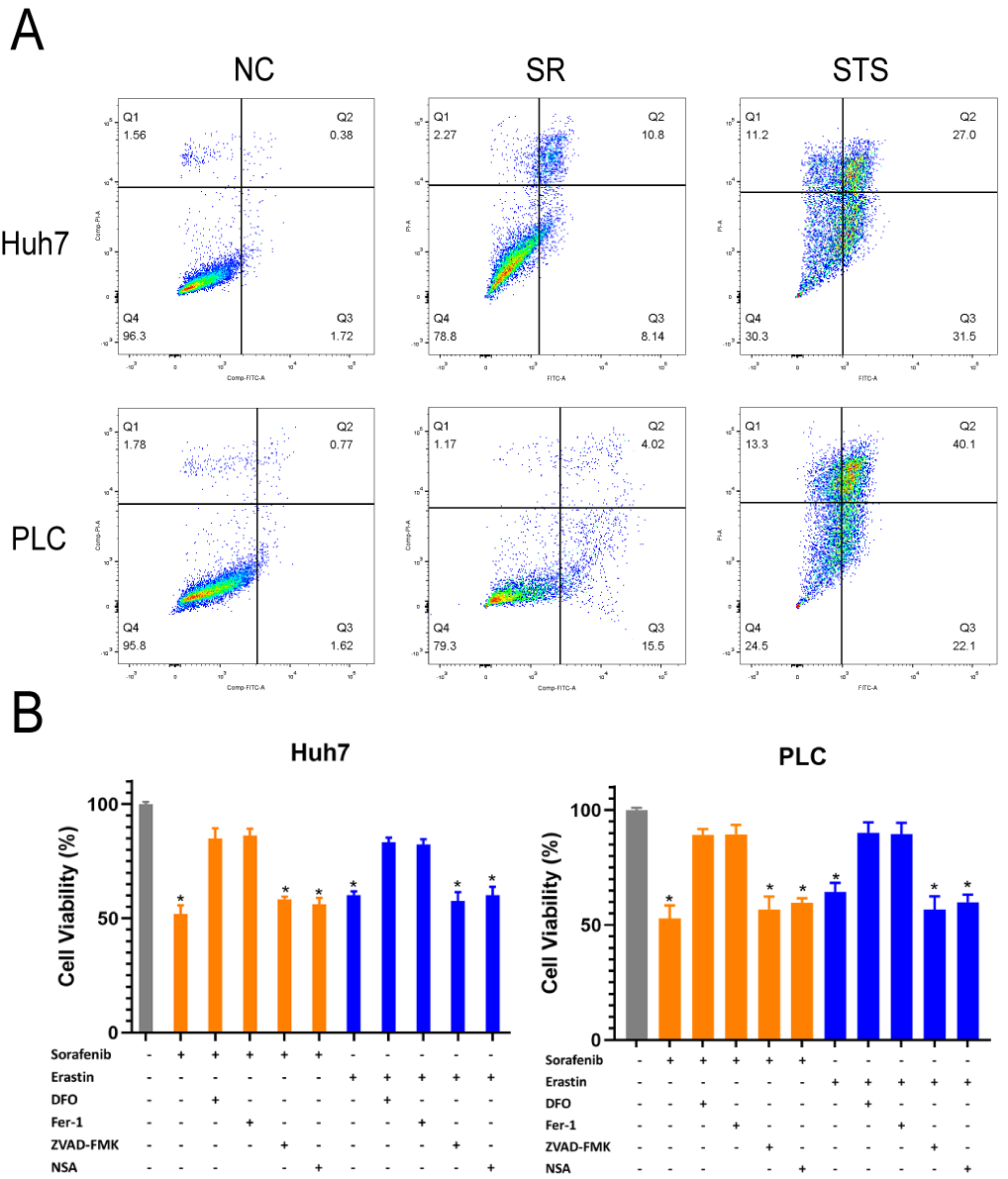

Supplement: Supplementary Figure 2 — (A) Flow cytometry was used to detect the apoptosis level of HCC cells under treatment with sorafenib (5 μmol) or STS (0.5 μmol). (B) CCK-8 method was used to detect the cell viability under treatment with sorafenib (5 μmol), erastin (5 μmol) with or without cell death inhibitors (Fer-1, 1 μmol; DFO, 200 μmol; ZVAD-FMK, 10 μmol) for 24 h (n=3, * P<0.05 versus NC group). [file Image_2.tif]

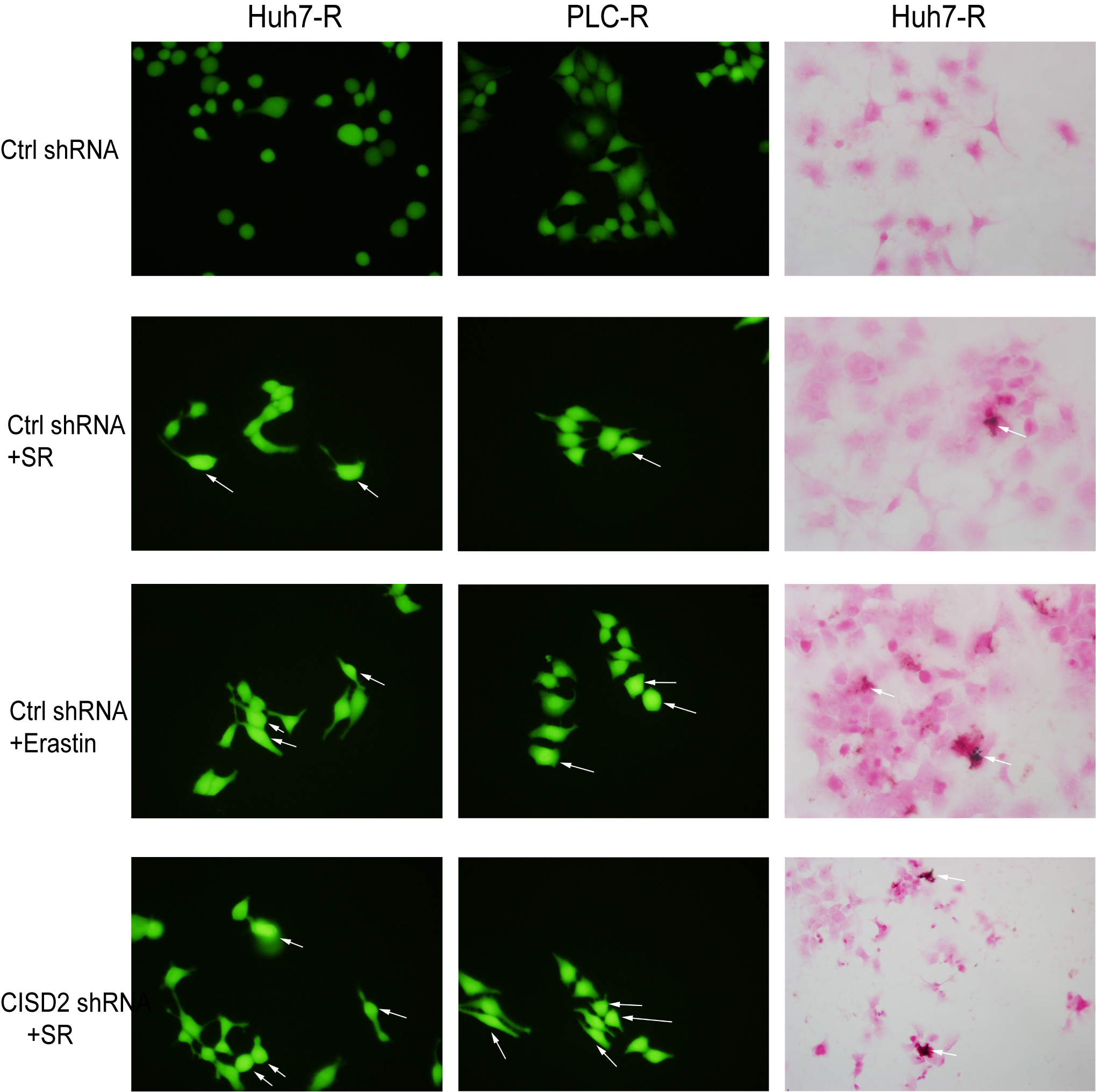

Supplement: Supplementary Figure 3 — Inverted fluorescence and Perls stain were used to detect the expression level of iron ions under treatment with sorafenib (10 μmol), erastin (10 μmol), or transfected CISD2 shRNA. [file Image_3.tif]
